# RBM4a-regulated splicing cascade modulates the differentiation and metabolic activities of brown adipocytes

**DOI:** 10.1038/srep20665

**Published:** 2016-02-09

**Authors:** Jung-Chun Lin, Yi-Han Lu, Yun-Ru Liu, Ying-Ju Lin

**Affiliations:** 1 School of Medical Laboratory Science and Biotechnology, College of Medical Science and Technology, Taipei Medical University, Taipei, Taiwan; 2 Joint Biobank, Office of Human Research, Taipei Medical University, Taipei, Taiwan; 3 School of Chinese Medicine, China Medical University, Taichung, Taiwan

## Abstract

RNA-binding motif protein 4a (RBM4a) reportedly reprograms splicing profiles of the *insulin receptor* (*IR*) and *myocyte enhancer factor 2C* (*MEF2C*) genes, facilitating the differentiation of brown adipocytes. Using an RNA-sequencing analysis, we first compared the gene expressing profiles between wild-type and *RBM4a*^−/−^ brown adipocytes. The ablation of RBM4a led to increases in the PTBP1, PTBP2 (nPTB), and Nova1 proteins, whereas elevated RBM4a reduced the expression of PTBP1 and PTBP2 proteins in brown adipocytes through an alternative splicing-coupled nonsense-mediated decay mechanism. Subsequently, RBM4a indirectly shortened the half-life of the *Nova1* transcript which was comparatively stable in the presence of PTBP2. RBM4a diminished the influence of PTBP2 in adipogenic development by reprogramming the splicing profiles of the *FGFR2* and *PKM* genes. These results constitute a mechanistic understanding of the RBM4a-modulated splicing cascade during the brown adipogenesis.

Alternative splicing (AS) constitutes a prevalent mechanism in expanding the genetic diversity of eukaryotic cells^1^. Approximately 90% of human genes generate more than one transcript by undergoing this meticulous process^2^,^3^. Spatiotemporal expression profiles of AS events control cell differentiation and specification^4^,^5^. The interplay between *trans*-factors and corresponding *cis*-elements within transcripts precisely manipulated tissue- and stage-specific splicing events^6^. Altering expression levels of splicing factors is an efficient and dominant mean to fine-tune alternative splicing profiles^7^,^8^. It is imperative to obtain comprehensive insights into AS events in the genome-wide era^9^.

Adipose tissue is an endocrine organ that participates in energy homeostasis. White and brown adipocytes (BAs) were identified based on their macroscopic appearance^10^. BAs dissipate fatty acids in the form of heat to maintain the body temperature, which implies its therapeutic potential for combating obesity^11^. The developmental process of BAs is still debated. Transcriptome analyses and lineage tracing suggest the existence of the same precursor cells for myocytes and BAs^12^. A BA-related protein network was uncovered, but the comprehensive mechanism is largely unknown^13^. AS constitutes a molecular mechanism which modulates adipocyte development. AS events, including of *IR* and *MEF2C* transcripts, encode adipocyte-specific isoforms which facilitate brown adipogenesis^14^,^15^. Interest is building in examining the AS-mediated mechanism involved in the development of BAs in terms of fat metabolism.

RNA-binding motif protein 4a (RBM4a) is a multifunctional protein that regulates various AS events in differentiating or malignant cells^16^,^17^,^18^. RBM4a was shown to reprogram the tissue-specific splicing profiles that facilitates the differentiation of myocytes and brown adipocytes^14^,^16^. An increase in RBM4a induces a relatively high level of the MEF2Cγ- protein which constitutes a feed-forward circuit toward upregulating RBM4a during brown adipogenesis^15^. In this study, results of the deep RNA-sequencing showed the differential gene expressions in *RBM4a*^−/−^ brown adipose tissues (BATs) compared to the wild type counterparts. The ablation in RBM4a with concomitant increases in the *Nova1*, *PTBP1*, and *PTBP2* transcripts was originally noted in BAs. Nova1 and PTBP2 were first demonstrated to be repressors of the development of BAs. Moreover, the RBM4a-regulated splicing cascade influenced the differentiating signaling and energy expenditure of pre-brown adipocytes.

## Results

### Differential gene expression in *RBM4a* ^−/−^ mice BATs

Two copies of *RBM4* (*RBM4a* and *RBM4b*) are organized similarly in terms of exons and introns, but are transcribed in opposite directions within the mouse genome^19^. The dominant expression and effect of RBM4a on the development of skeletal muscle and brown adipose tissues was documented^14^,^15^,^16^. To obtain comprehensive insights into the physiological functions and regulation of the *RBM4a* gene during brown adipogenesis, deep RNA sequencing were performed with RNAs prepared from *RBM4a*^−/−^ or the wild-type (WT) BATs. Table 1A showed the summary statistics that the numbers of mapped reads were close in different samples within independent analyses (*n* = 4). The transcript levels of 626 genes were relatively abundant in WT BATs compared to the *RBM4a*^−/−^ counterparts, whereas 175genes exhibited relatively high transcript levels in *RBM4a*^−/−^ BATs (Table 1B). The ablation of RBM4a led to changes in gene expressions as partially shown in Table 2. Relatively abundant *PGC-1α*, *PGC-1β*, and *UCP1* transcripts were noted in WT BATs compared to *RBM4a*^−/−^ counterparts. Ablation of RBM4a resulted in the relatively high levels of two paralogous transcripts, *PTBP1* and *PTBP2*, which were first revealed in BAs. Intriguingly, RBM4a had an opposite effect on expressions of other paralogous genes, *Nova1* and *Nova2*, which widely participate in the development of neuronal cells^20^. Although the compensative expression of *RBM4b* was observed in *RBM4a*^−/−^ BATs, the differential expression profiles suggested a specific and dominant role played by RBM4a in brown adipogenesis.

### Differential expression profiles of splicing factors in WT and *RBM4a* ^−/−^ BAs

**Figure 1:**
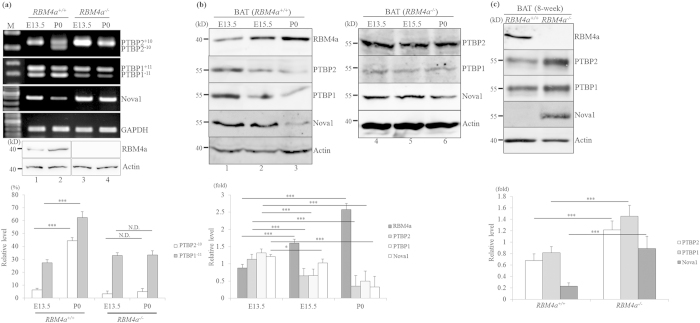
Differential expression profiles of RBM4a, PTBP1, PTBP2, and Nova1 during brown adipogenesis. (**a**) Total RNA and protein extracted from embryonic day (E) 13.5 and postnatal day 0 (P0) *RBM4a*^+/+^ (wild-type) or *RBM4a*^−/−^ interscapular brown adipose tissues were subjected to an RT-PCR and immunoblot assay using specific primer sets and antibodies. (**b**) Tissue lysates extracted from embryonic day (E) 13.5, 15.5 and postnatal day 0 (P0) *RBM4a*^+/+^ (wild-type) or *RBM4a*^−/−^ interscapular brown adipose tissues were subjected to immunoblot analyses with the indicated antibodies. (**c**) Tissue lysates extracted from brown adipose tissues of WT or *RBM4a*^−/−^ adult mice were subjected to an immunoblot assay with the indicated antibodies. The gels or blots showed in this figure were run under the same conditions and not artificially manipulated. The bar graph represents relative levels of the indicated proteins or transcripts in three independent experiments using TotalLab Quant Software (*p < 0.05; **p < 0.01; ***p < 0.005).

To validate the analytic results of RNA sequencing, total RNAs prepared from *RBM4a*^−/−^ BATs or the WT counterparts were subjected to RT-PCR assays. Figure 1 showed the increases in *PTBP2*^*−10*^ and *PTBP1*^*−11*^ transcripts, the nonsense-mediated decay (NMD) substrates^21^, which were noted during the development of BATs (Fig. 1a, lanes 1 and 2). The relative levels of *PTBP2*^+*10*^ and *PTBP1*^+*11*^ transcripts were sustained in *RBM4a*^−/−^ embryonic (E13.5) and postnatal (P0) BATs (lanes 3 and 4) compared to that of WT littermates. The expression of *Nova1* transcripts was unchanged in embryonic and postnatal *RBM4a*^−/−^ BATs, whereas a gradual decrease in *Nova1* transcripts was observed during the development of BATs (Fig. 1a). Immunoblotting assays showed the gradual increase in the RBM4a protein with concomitant decreases in expressions of PTBP2, PTBP1, and Nova1 proteins (Fig. 1b, lanes 1–3) which were relevant to the transcript levels. Ablation of RBM4a resulted in the unchanged expression profiles of PTBP2, PTBP1, and Nova1 proteins in embryonic and postnatal BATs (lanes 4–6). Differential expressions of PTBP2, PTBP1 and Nova1 proteins were consistently noted in BATs dissected from adult *RBM4a*^−/−^mice and WT littermates (Fig. 1c). Therefore, unlike RBM4a, PTB proteins and Nova1 may possess distinct effect toward the development of BATs.

### RBM4a modulates expression profiles of PTBP2, PTBP1, and Nova1 in preadipocytes

**Figure 2:**
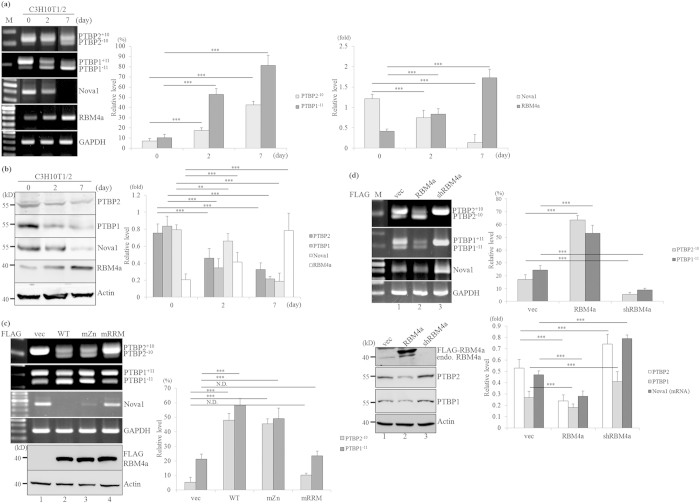
RBM4a alters the PTBP1, PTBP2, and Nova1 protein levels during brown adipogenesis. (**a**) Total RNAs and (**b**) cell extracts isolated from C3H10T1/2 cells cultured in growth medium (0 day) and differentiating medium (for 2 and 7 days) were subjected to RT-PCR, qRT-PCR and immunoblotting assays using specific primer sets and antibodies. (**c**) Total RNAs and cell extracts extracted from C3H10T1/2 cells that overexpressed RBM4a and the derived mutants were subjected to RT-PCR and immunoblotting assays using specific primer sets and antibodies. (**d**) Total RNAs and cell extracts extracted from RBM4a overexpressing or targeting C3H10T1/2 cells were subjected to RT-PCR, qRT-PCR and immunoblotting assays as previously described. The gels or blots showed in this figure were run under the same conditions and not artificially manipulated. The bar graph represents relative levels of the indicated proteins or transcripts in three independent experiments using TotalLab Quant Software (*p < 0.05; **p < 0.01; ***p < 0.005).

*In vitro* differentiation of C3H10T1/2 cells was subsequently conducted to confirm previous observations. Using RT-PCR (Fig. 2a, left) and quantitative(q)RT-PCR analyses (Fig. 2a, right), the gradual increase in the endogenous *RBM4a* with concomitant decreases in the *PTBP2*^+*10*^, *PTBP1*^+*11*^, and *Nova1* transcripts was observed during the differentiation of C3H10T1/2 cells. The immunoblotting assay indicated the relevant expression of PTBP2, PTBP1, RBM4a, and Nova1 to the transcript profiles during the differentiating process (Fig. 2b). The direct binding of RBM4a to the CU-elements within *PTBP1* exon 11and flanking introns led to *PTBP1* exon 11 skipping in myoblast cells^16^. The presence of overexpressing RBM4a or the derived zinc knuckle-mutant (mZn) containing the authentic RNA recognition motifs (RRMs) reduced the relative level of *PTBP2*^+*10*^, *PTBP1*^+*11*^ and *Nova1* transcripts (Fig. 2c, lanes 1–3). In contrast, the abundances of *PTBP2*^+*10*^, *PTBP1*^+*11*^, and *Nova1* transcripts showed no response to the RRMs-mutant RBM4a containing the authentic zinc knuckle motif which potentially involved in DNA/protein-protein interaction^22^ (lane 4). Immunoblotting analyses clarified that distinct effects of RBM4a variants on *PTBP2*, *PTBP1,* and *Nova1* expression relied on their biological activities since the protein levels were equal (Fig. 2c, RBM4s). As shown in Fig. 2d, increase in the *PTBP2*^+*10*^, *PTBP1*^+*11*^and *Nova1* transcripts was noted in the RBM4a targeting cells compared to the empty vector-transfected cells (upper panel, lanes 1 and 3). The alteration of RBM4a manipulated the protein levels of PTBP1/2, consistently reflecting the transcript profiles (lower panel). These results indicated the hierarchical role of RBM4a in overriding the crossregulation between *PTBP1* and *PTBP2* genes^16^. The lost influence of RRMs-mutant on *PTBP2*, *PTBP1,* and *Nova1* transcripts implied that RBM4a mainly modulated the expression of these genes through post-transcriptional regulation.

### PTBP2 stabilizes *Nova1* transcripts by binding to its 3′-UTR

**Figure 3:**
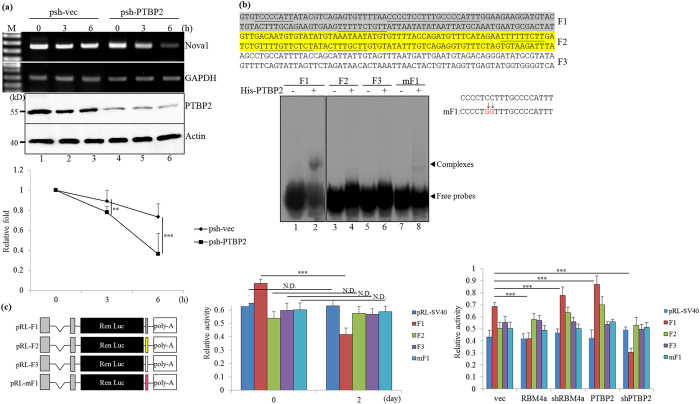
PTBP2 stabilizes the *Nova1* transcript via directly binding to its 3′ untranslated region (UTR). (**a**) Empty vector- or PTBP2 targeting vector-transfected C3H10T1/2 cells were mock-treated or treated with actinomycin D. Total RNA and cell extract prepared at indicated time points were subjected to RT-PCR, qRT-PCR, and immunoblotting assays with specific primer sets and antibodies. (**b**) The diagram presents the sequence of the proximal *Nova1* 3′ UTR. The mock eluate or recombinant His-tagged PTBP2 protein (2 μg) eluted from the Ni2+ agarose resin was incubated with 10 nM DIG-labeled probes. The mixtures were fractionated on an 8% native acrylamide gel and transferred to a nylon membrane. The membrane was probed using the horseradish peroxidase-conjugated anti-DIG Fab fragment. The gels or blots showed in this figure were run under the same conditions and not artificially manipulated. (**c**) The scheme shows the Renilla luciferase reporter containing the distinct fragment within the *Nova1* 3′ UTR. The intact pRL-*Nova1* 3′ UTR reporters or the derived mutant were transfected into C3H10T1/2 cells cultured under distinct conditions or cotransfected with the expressing or targeting vectors and the pGL3-basic reference vector into C3H10T1/2 cells. The luciferase assays were performed as described under “Materials and methods” and the bar graphs show relative renilla luciferase activity in three independent experiments. The statistical analyses showed the convincing difference in the activity of F1 reporter, but not other reporters in distinct experiment groups (*p < 0.05; **p < 0.01; ***p < 0.005).

Nova1 and Nova2 genes are homologous and phylogenetically conserved, but substantially differ in their localization and 3′-UTR sequences. The long 3′-UTR of Nova1 is highly conserved in mammals, whereas the Nova2 3′-UTR is relatively diverse among species^23^. The interaction between nELAV and Nova1 3′-UTR constituted a post-transcriptional mechanism for stabilizing Nova1 transcripts^24^. In addition, PTBP2 was reported to enhance the stability of *phosphoglycerate kinase 2* and *inducible nitric oxide synthase* transcripts by binding to its 3′-UTRs^25^. We therefore wondered whether PTBP2 participated in the 3′-UTR-mediated regulation of *Nova1* transcripts. The RT-PCR and qPCR results indicated that around 80% of *Nova1* transcripts were left with treatment of actinomycin D for 6 h (Fig. 3a, lanes 1 and 3; diamond), but only 36% of the *Nova1* transcripts were observed in *PTBP2*-knockdown cells with the same treatment (lanes 4 and 6; square). This result implied the potential influence of PTBP2 on the stability of *Nova1* transcripts. According to the binding tendency of the PTB proteins toward UCUU or CUCUCU motif, the CU-rich elements were noted within the proximal region of the *Nova1* 3′-UTR (Fig. 3b, underlined). To determine putative binding site of the PTBP2, the gel-shift assays were performed by incubating the *in vitro* transcribed and DIG-labeled RNA probes (F1, F2 and F3) with the His-tagged PTBP2. Only the F1 (Fig. 3b, lower, lane 2), but not F2 or F3 probe (lanes 4 and 6), formed ribonucleoprotein complexes with the recombinant PTBP2 protein. The interaction between the F1 probe and recombinant PTBP2 was abolished with guanine nucleotide substitutions within the UCCU motif (mF1, CC to GG, lane 8). The functional relevance of the PTBP2 binding motif (UCCU) was examined by introducing the renilla luciferase reporter containing F1, F2, F3, or mutant F1 fragment into C3H10T1/2 cells. The reduced activity of the F1 fragment-containing reporter was noted in differentiating C3H10T1/2 cells (Fig. 3c, middle, brick color), whereas the activities of other reporters were unchanged in proliferating and differentiating cells (Fig. 3c, middle). Moreover, the statistical analyses of *in vivo* translation assay showed that only the F1 element-containing reporter, but not the other reporters, exhibited convincing responses to the RBM4a and PTBP2 protein levels. Overexpressing PTBP2 or short hairpin (sh)RNA-mediated ablation of RBM4a preferentially enhanced the activity of the F1-containing reporter. Overexpressing RBM4a or shRNA-mediated knockdown of PTBP2 inversely led to reduced activity of the F1-containing reporter (Fig. 3c, right, brick red). Activities of the Renilla reporters that contained the F2, F3, or mF1 fragment were not altered with change of PTBP2 and RBM4a expression (Fig. 3c, right). These results identified RBM4a- PTBP2interplay as a novel posttranscriptional mechanism of the *Nova1* expression in BAs.

### PTBP2 and Nova1 function as brown-adipogenic repressors

**Figure 4:**
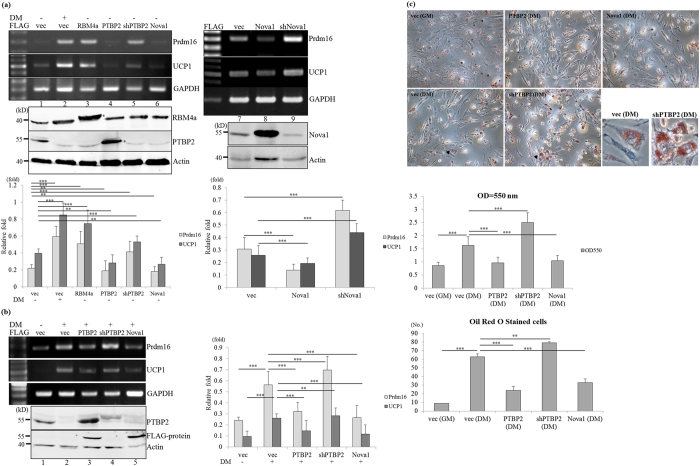
RBM4a, PTBP2, and Nova1 possess differential effects on brown adipogenesis. (**a**) C3H10T1/2 cells were transfected with the expression vectors of RBM4a, PTBP2, Nova1, or targeting vectors of PTBP2 and Nova1. Total RNAs and cell extracts were extracted from the transfectants cultured in growth or differentiating medium, followed by RT-PCR, qRT-PCR and immunoblotting analyses. (**b**) C3H10T1/2 cells were transfected with expressing vectors of PTBP2 and Nova1, or targeting vectors of PTBP2 and cultured in differentiating medium 24 h post-transfection. After 48 h, total RNAs and cell extracts were isolated from the transfectants and subjected to RT-PCR, qRT-PCR and immunoblotting analyses. The bar graph presents results of the qRT-PCR in three independent experiments. The gels showed in this figure were run under the same conditions and not artificially manipulated. (**c**) Parallel experiments were performed as described in the last section and then subjected to oil-red-O staining. The bar graph shows the spectrophotometric analysis of the extracted oil-red-O optical density (OD) at 550 nm and numbers of oil-red-O-stained cells in 100 cells (*p < 0.05; **p < 0.01; ***p < 0.005).

The gradual decreases in PTBP2and Nova1 proteins throughout the brown adipogenesis (Figs 1b and 2a) prompted further investigation to their potential effects on BAs differentiation. RT-PCR (Fig. 4A, left) and qRT-PCR results (bar graph) showed the reproducible results that transient overexpression of FLAG-tagged PTBP2and Nova1 proteins reduced the BA-specific factors, *Prdm16* and *UCP1* transcripts, compared to the vector-transfected cells (lanes 1, 4 and 6). In contrast, shRNA-induced knockdown of PTBP2 and Nova1increased *Prdm16* and *UCP1* transcript levels (lanes 5 and 9) as observed in the differentiating or RBM4a-overexpressing cells (lanes 2 and 3). The presence of overexpressing PTBP2and Nova1 proteins counteracted the effect of differentiating medium on inducing *Prdm16* and *UCP1* transcripts compared to the empty vector-transfected cells (Fig. 4b, lanes 2, 3 and 5), whereas shRNA-mediated knockdown of PTBP2strengthened the effect of the differentiating condition on *Prdm16* and *UCP1* expression (Fig. 4b, lane 4). qRT-PCR analyses showed the convincing expression profiles of *Prdm16* and *UCP1* in parallel experiments (Fig. 4b, bar chart). Oil-red O staining results identically illustrated that ablation of PTBP2enhanced the lipid accumulation in differentiating C3H10T1/2 cells (Fig. 4c, shPTBP2), whereas PTBP2- or Nova1-overexpressing cells exhibited less lipid accumulation compared to empty vector-transfected cells cultured in the differentiating condition. Enlarged images indicated that differentiating medium mediated more lipid accumulation in PTBP2-knockdown cells compared to the empty vector-transfected cells (Fig. 4c, arrowhead). Imaging results were quantitatively evaluated in terms of extracted oil-red-O using spectrophotometric analysis (Fig. 4c, middle) and counting of oil-red O-stained cells among 100 cells in total (Fig. 4c, lower). The quantitative approaches indicated the influence of overexpressing PTBP2, Nova1 or PTBP2 ablation on the metabolic signature of differentiating C3H10T1/2 cells. These results identified the repressive effect of PTBP2 and Nova1 on the BAs development and metabolism.

### RBM4a-PTBP2 network manipulates BAT-related splicing events

**Figure 5:**
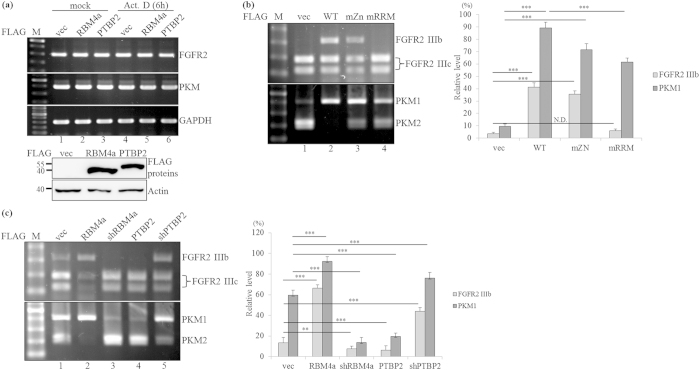
RBM4a and PTBP2 proteins exhibit differential effects on splicing profiles of the *FGFR2* and *PKM* genes. (**a**) C3H10T1/2 cells were transfected with the expression vectors of RBM4a or PTBP2. Total RNAs and cell extracts extracted from the mock or actinomycin D-treated cells were subjected to an RT-PCR and immunoblotting analysis. (**b**) C3H10T1/2 cells were transfected with the expression vectors of RBM4a and derived mutants. Total RNAs extracted from the transfectants were subjected to an RT-PCR analysis, followed by digestion with restriction enzymes. (**c**) Total RNAs were isolated from C3H10T1/2 cells transfected with the expression or targeting vectors of RBM4a and PTBP2, followed by an RT-PCR assay and enzyme digestion. The gels showed in this figure were run under the same conditions and not artificially manipulated.The bar graph presents relative levels of PCR-amplified transcripts in three independent experiments using TotalLab Quant Software (*p < 0.05; ** *p* < 0.01; ***p < 0.005).

Around 74% similarity between the RRMs sequence of PTBP2 and PTBP1 implies that PTBP2 possibly exerts a similar specificity to PTB-regulated splicing events, including *FGFR2* and *PKM*, which played pivotal role in the differentiating signal and metabolic function of BAs^26^,^27^,^28^. Overexpression of PTBP1 enhanced the relative levels of *FGFR2 IIIc* and *PKM2* in C3H10T1/2 cells, whereas the relatively high levels of *FGFR2 IIIb* and *PKM1* transcripts were noted in the PTBP1 targeting cells (Supplementary Fig. 1). We therefore wondered whether RBM4a- PTBP2 interplay manipulated the splicing profile of the *FGFR2* and *PKM* genes in BAs. The expression profiles (lanes 1–3) and stabilities (lanes 4–6) of *FGFR2* and *PKM* transcripts remained sustained with the overexpression of RBM4a and PTBP2 (Fig. 5a). RT-PCR results showed the predominant expression of *FGFR2 IIIc* and *PKM2* transcripts in C3H10T1/2 cells as other progenitor cells (Fig. 5b, lane 1). Overexpressing RBM4a and the derived mZn mutant profoundly enhanced the relative level of *FGFR2 IIIb* (Fig. 5b, upper, lanes 2 and 3), whereas mRRMs mutant completely lost the activity (lane 4). Similarly, the relative level of *PKM1* transcripts was elevated in the presence of overexpressing RBM4a and the derived mZn mutant (Fig. 5b, lower, lanes 2 and 3), whereas the mutations within RRMs diminished this phenomenon (Fig. 5b, lower, lane 4). The relatively high levels of the *FGFR2 IIIb* and *PKM1* transcripts were noted in PTBP2-knockdown C3H10T1/2 cells compared to empty vector-transfected cells (Fig. 5c, lanes 1 and 5). In contrast, overexpressing PTBP2 or RBM4a-knockdown enhanced the relative levels of the *FGFR2 IIIc* and *PKM2* transcripts (lanes 3 and 4). These results indicated the differential effect of RBM4a and PTBP2 on programming the splicing profiles of *FGFR2* and *PKM* genes in BAs.

### The RBM4a-regulated splicing event modulates the brown-adipogenic signaling pathway

**Figure 6:**
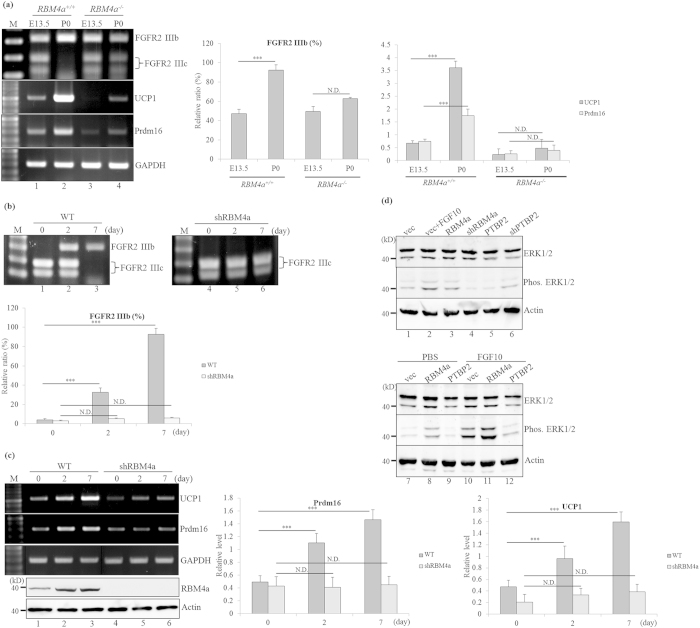
RBM4a-modulated splicing profile of *FGFR2* correlates with brown adipogenesis-related signaling. (**a**) Total RNA extracted from the embryonic day (E) 13.5, and postnatal day 0 (P0) *RBM4a*^+/+^ (wild-type) or *RBM4a*^−/−^ interscapular brown adipose tissues was subjected to RT-PCR, enzyme digestion and qRT-PCR analyses. (**b,c**) Total RNAs and cell extract were isolated from the proliferating and differentiating cells at different time points, followed by RT-PCR, enzyme digestion, qRT-PCR and immunoblotting assay with specific primer sets and antibodies. The bar graph presents relative levels of the PCR-amplified transcripts (*FGFR2*) or a qRT-PCR analysis (*Ucp1* and *Prddm16*) using TotalLab Quant Software (*p < 0.05; **p < 0.01; ***p < 0.005). (**d**) C3H10T1/2 cells were transfected with an empty vector, expression vectors, or targeting vectors of RBM4a and PTBP2. Before being harvested, transfected cells were mock-treated or treated with FGF10 for 6 h. Cell extracts were isolated from transfectants, followed by immunoblotting assays with anti-ERK1/2, anti-phospho ERK1/2, and anti-Actin antibodies. The gels or blots showed in this figure were run under the same conditions and not artificially manipulated.

FGF10-FGFR2 IIIb interaction activated the downstream ERK1/2 and p38 MAPK pathway, which enhanced *UCP1* transcripts and β-oxidation of fatty acids in BATs^29^,^30^. Controversially, upregulated FGFR2 expression was reported to interfere with adipocytic differentiation of C3H10T1/2 cells^31^. We thus wondered whether alternative splicing constituted a spatiotemporal mechanism in manipulating the effect of FGFR2 on the downstream signal. Using an RT-PCR assay, the predominant expression of *FGFR2 IIIb* transcripts was noted in postnatal BATs (Fig. 6a, lane 2), whereas the relative level of *FGFR2 IIIc* transcripts were close during the development of *RBM4a*^−/−^BATs (lanes 3 and 4). Synchronous increases in *UCP1*and *Prdm16* transcripts (lane 2) implied their relevance between FGFR2 IIIb-mediated signaling. qRT-PCR result indicated the close expression of *UCP1* and *Prdm16* transcripts with an unchanged splicing profile of *FGFR2* in embryonic and postnatal *RBM4a*^−/−^ BATs (Fig. 6a, right bar chart). This result was consistently reproduced in response to differentiation of C3H10T1/2 cells. The differentiating condition drove the isoform change of the *FGFR2* gene in C3H10T1/2 cells (Fig. 6b, lanes 1–3), whereas the splicing profile of *FGFR2* remained unchanged throughout the differentiation of RBM4a-knockdown cells (lanes 4–6). RT-PCR and qRT-PCR results showed the gradual increases in *UCP1*and *Prdm16* transcripts during the differentiation of C3H10T1/2 cells (Fig. 6c, lanes 1–3), but RBM4a silencing substantially abolished this phenomenon (lanes 4–6; qRT-PCR, bar chart).

We next validated the influence of RBM4a- PTBP2 interplay on brown adipogenesis-related ERK1/2 signaling. Using immunoblotting assays, we noted that FGF10 treatment, RBM4a overexpression, or PTBP2 knockdown executed similar effects of inducing the phosphorylation of ERK1/2 compared to empty vector-transfected cells (Fig. 6d, lanes 1–3 and 6). RBM4a-knockdown or PTBP2-overexpressing cells inversely exhibited less phosphorylated ERK1/2 proteins (lanes 4 and 5). Moreover, overexpressing RBM4a synergized the FGF10-induced phosphorylation of ERK1/2 (lane 11), whereas overexpressing PTBP2 diminished the effect of FGF10 on ERK1/2 activation (lane 12). These results revealed the influence of RBM4a-regulated splicing cascade on manipulating the BA-related signaling.

### RBM4 and PTBP2 exert opposite effects on the energy expenditure of BAs

**Figure 7:**
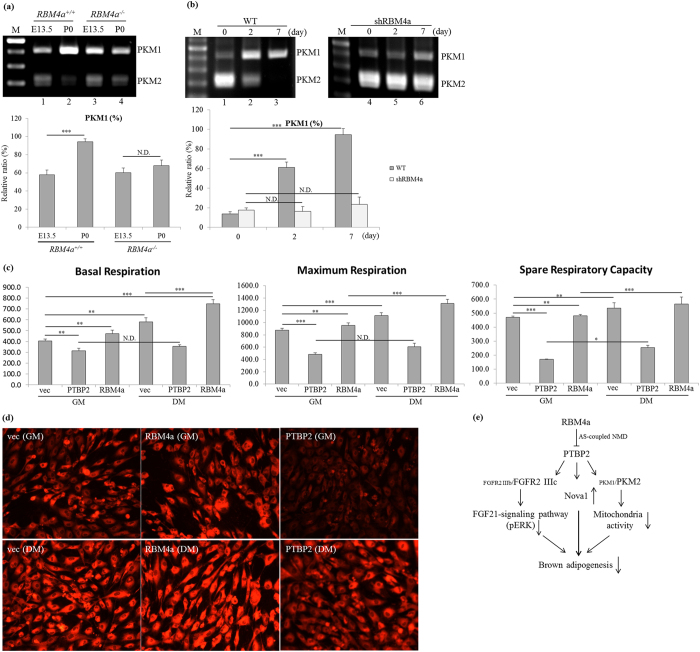
RBM4a-regulated splicing profile of *PKM* gene modulates the mitochondrial activity of brown adipocytes. (**a**) Total RNA extracted from embryonic day (E) 13.5 and postnatal day 0 (P0) *RBM4a*^+/+^ (wild-type) or *RBM4a*^−/−^ interscapular brown adipose tissues were subjected to RT-PCR analyses and enzyme digestion. (**b**) Total RNAs and cell extract were isolated from proliferating and differentiating cells at different time points, followed by RT-PCR assay and enzyme digestion. The gels showed in this figure were run under the same conditions and not artificially manipulated. The bar graph presents relative levels of the PCR-amplified transcripts using TotalLab Quant Software (*p < 0.05; **p < 0.01; ***p < 0.005). (**c**) C3H10T1/2 cells were transfected with expression vectors of RBM4a or PTBP2 for 24 h. Transfectants were then cultured in growth medium or differentiating medium for an additional 48 h. The bar graph presents the basal and maximal oxygen consumption rates and spare respiratory capacity that were measured using an XF20 Bioanalyzer (*n* = 4). (**d**) Parallel experiments were performed as described in the last section. Mitochondrial content were visualized by epifluorescence in living cells with the Mitotracker Red FM dye. (**e**) The RBM4a-regulated splicing cascade correlated with brown adipogenesis. Elevated RBM4a induced alternative splicing-coupled nonsense-mediated decay toward *PTBP1*/*2* transcripts in differentiating brown adipocytes. The interplay between RBM4a and PTBP2 programmed the splicing profiles of the *FGFR2* and *PKM1* genes which manipulated the differentiation signaling and energy expenditure of brown adipocytes.

The expression profiles of *PKM* gene is spatiotemporally modulated by an AS mechanism^32^. PKM2 is an embryonic or carcinogenic isoform, whereas PKM1 is mainly expressed by differentiated or energy-expenditure cells, including BAs^33^,^34^. As shown in Fig. 7a, the gradual increase in *PKM1* transcript as that of *FGFR2 IIIb* was only observed in WT BATs (lanes 1and 2), but not in the *RBM4a*^−/−^BATs (lanes 3 and 4). Similar results were reproducible in a culture system of the dominant expression of the *PKM2* transcript which gradually shifted to *PKM1* transcripts during the differentiation of C3H10T1/2 cells (Fig. 7b, lanes 1–3). RBM4a-knockdown cells exhibited a relatively high level of the *PKM2* transcript throughout the differentiating process (lanes 4–6). The results of bioenergy analyses showed that RBM4a-overexpressing cells exhibited higher basal oxygen consumption rate (OCR) compared to empty vector-transfected cells, whereas overexpressing PTBP2 inversely reduced the basal OCR of proliferating cells (Fig. 7c, basal respiration, GM). Moreover, the additive upregulation of basal OCR was noted in RBM4a-overexpressing cells maintained in differentiating medium, but no substantial difference in the basal OCR was observed in PTBP2-overexpressing cells under the same condition (Fig. 7c, basal respiration, DM). Similarly, the maximal and spare respiratory capacities remained unchanged in PTBP2-overexpressing cells cultured in proliferating or differentiating medium (Fig. 7c, maximum respiration and respiratory capacity). Figure 7d showed that the PTBP2-overexpressing cells exhibited less mitochondrial biogenesis than that of vector-transfected cells cultured in proliferating or differentiating medium. In contrast, the relatively elevated mitochondriogenesis was noted in the RBM4a-overexpressing cells cultured under the same conditions. These results indicated the opposite effects of RBM4a and PTBP2 on the metabolic activities of BAs which were relevant to *PKM* splicing profile and mitochondriogenesis.

## Discussion

Posttranscriptional controls constitute complex mechanisms in programming expression profiles or fine-tuning biological activities of tissue-specific factors^35^. Deep RNA-sequencing provides an advance approach to establish a global view of transcript profiles in a spatiotemporal manner^36^. Herein, we performed a deep RNA-sequencing to evaluate the influence of RBM4a on transcript profiles in BAs. The results showed that ablation of RBM4a profoundly affected BA-related genes expressions which subsequently influenced the development or function of BAs.

RBM4a facilitated brown adipogenesis by programming a subset of alternative splicing events^14^,^15^. In this study, the *RBM4a*^−/−^ BATs exhibited upregulated levels of *PTBP2* and *Nova1* transcripts (Fig. 1) which functioned as repressors toward both the development and metabolic signature of BAs (Figs 4 and 7). Overexpressing PTBP2 and Nova1restricted the development of BA-like progenitor cells, and the differentiating process was initiated with a decline in their expression. Similarly, PTBP2-regulated splicing events maintained the biological signature of neuronal progenitor cells and a gradual reduction in PTBP2 was noted during the development of mature neurons^21^,^37^. Accordingly, PTBP2-programmed splicing profiles may globally silence the inaccurate differentiation of progenitor cells more than maintaining their biological features. PTBP1 was reported to constitute an autoregulatory feedback circuit by enhancing the relative level of *PTBP1*^*−11*^ and *PTBP2*^*-10*^ transcripts^38^. Overexpressing PTBP2 increased its abundance by enhancing the utilization of its exon 10, which partially neutralized the repressive effect of elevated PTBP1 in colorectal cancer cells^39^. Nevertheless, RBM4a played a hierarchical role that overrode the crossregulation between PTB proteins in different cells. The RBM4a- PTBP2 interplay constituted a regulatory mechanism that widely modulated the brown adipogenesis. Identification of more RBM4a-, PTBP2- and Nova1-specific splicing event can be applied in emphasizing the inference.

Besides being a well-known splicing factor, PTBP2 functioned as a multifunctional RNA-binding protein involved in mRNA localization, IRES-mediated translation, and mRNA stability^40^. Although *in vitro* studies suggested the binding tendency of PTB proteins towards UCUU or CUCUCU motifs, the interactions between PTB proteins and candidates do not specifically rely on the consensus sequence^41^. For instance, PTBP2 protein enhanced the stability of *pgk2* transcripts by binding to the non-consensus CU-elements within the *pgk2* 3′ UTR^25^. Multiple CU-rich elements shared different sequences additively strengthened the binding of PTBP2 to the *pgk2* 3′ UTR. In our study, the *in vitro* binding and functional assay identified UCCU motif within *Nova1* 3′ UTR as a binding site of PTBP2 protein (Fig. 3). The presence of four RRMs was proposed as a molecular mechanism that largely expanded the binding tendency of the PTB proteins^42^. Herein, we provide another example regarding the effect of the PTBP2 protein on the 3′UTR-mediated gene regulation

As shown in Table 2, complementary expression profiles of the Nova1 and Nova2 proteins were revealed in *RBM4a*^−/−^ BATs compared to the WT littermates. *Nova1* and *Nova2* genes are phylogenetically conserved, but their 3′ UTR sequences greatly differ. The highly conserved *Nova1* 3′ UTR among distinct species suggests that 3′ UTR-mediated regulation may contribute to differential expressions of Nova 1 protein in distinct tissues or particular stage. PTBP2 was demonstrated to be a novel regulator in stabilizing *Nova1* transcripts by binding to the UCCU motif within its 3′ UTR in our work. The repressive effects of PTBP2 and Nova1 on the development and functioning of BAs were first revealed. It would be interesting to further evaluate the influence of Nova2, the relatively high expression of which was noted in ordinary BATs compared to *RBM4a*^−/−^ BATs. To bring the comprehensive insight into the mechanism underlying the differentiation of BAs, the specific candidate of RBM4a, PTBP2, and Nova family proteins in BAs is worthy of further identification by using high-throughput approaches.

FGFR2 isoforms are spatiotemporally generated through AS mechanisms^43^. The abundant level of FGFR2 IIIc was noted in mature white adipocytes^28^. Adipocyte-restricted *FGFR2*^−/−^ mice exhibited the impaired hypertrophy of white adipocytes and reduced plasma fatty acid, implying its potential influence on adipogenesis and lipid metabolism^28^. Interestingly, the overexpressing FGFR2 IIIc decreased the adipocytic differentiation of C3H10T1/2 cells which were considered as the progenitors of BAs^31^. Herein, we first revealed the gradual increase in the *FGFR2 IIIb* transcript during the differentiation of C3H10T1/2 cells and mice BATs (Fig. 6A and B). The effect of FGF10-FGFR2 IIIb interplay on ERKs phosphorylation indicated its potential function on a brown adipogenesis-related signaling pathway^29^. Therefore, RBM4a-induced increase in the relatively high level of *FGFR2 IIIb* may constitute a novel mechanism in promoting the brown adipogenesis. In addition, *PKM* gene encodes a constitutively active PKM1 isoform in differentiated cells and an embryonic PKM2 isoform which participated in the transition from aerobic respiration to anaerobic glycolysis^34^. The increased *PKM2* mRNA was noted in the NIH3T3-L1 cells with the insulin treatment^44^, whereas the predominant expression of *PKM1* transcripts was observed in BATs^45^. Nevertheless, the splicing profiles of *PKM* transcripts during the development of BATs were first revealed in this study (Fig. 7A and B). The splicing patterns of *FGFR2* and *PKM* genes were reprogrammed with the expression profiles of splicing factors, including RBM4a, PTB proteins (Fig 5B,C). The molecular mechanisms involved in the splicing of *FGFR2* and *PKM* genes were worthy of further investigation, which may bring a new insight into the differentiation of BAs.

In conclusion, we first correlated the regulation of two neuronal-specific splicing factors, PTBP2 and Nova1, within RBM4a-modulated posttranscriptional control in BAs. The relatively high levels of PTBP2 and Nova1 in *RBM4a*^−/−^ BATs suggested their repressive effects on brown adipogenesis. Identification of PTBP2 as a posttranscriptional regulator of Nova1 constituted a novel molecular mechanism for repressing the development of BATs. We also revealed the RBM4a-modulated splicing cascades that widely involved in the differentiation-related signaling and metabolic signature of BAs. Our results continuously elucidated the role that RBM4a plays in brown adipogenesis.

## Methods

### Ethics statement in animal research

All experiments and animal care were performed in accordance with the relevant guidelines and regulations. This study was approved according to the recommendations of the Guide of the Institutional Animal Care and Use Committee at Taipei Medical University under approved NO. LAC-2013-0208. All efforts were made to minimize animal suffering.

### Mice dissection

Male *RBM4a*^−/−^ mice were generated as previously described^19^. Adult mice were fed a regular diet for 8 weeks. After being euthanized, interscapular fat tissues were collected from adult and embryonic mice, weighed, and immediately frozen until the RNA and proteins were extracted.

### RNA extraction, complementary (c)DNA library construction, and sequencing

Total RNA was extracted using the PureLink RNA mini kit (Invitrogen, Camarillo, CA, USA) according to the manufacturer’s protocol. The RNA quality was first evaluated using agarose gel electrophoresis and staining with ethidium bromide (EB). The integrity and quantity of the RNA were further assessed using an Agilent 2100 Bioanalyzer (Agilent Technologies, Redwood, CA, USA). The total RNA with a high integrity number (RIN > 8.0) was subjected to library construction. In total, 8 μg of RNA per sample was applied to construct three cDNA libraries using the NEBNext Ultra RNA Library Prep Kit from Illumina (NEB, Ipswich, MA, USA) according to the manufacturer’s instructions. In brief, poly(A) messenger (m)RNA was enriched using oligo(dT)-attached magnetic beads. The poly(A) mRNA was next cleaved into small fragments using divalent cations in NEBNext first-strand synthesis reaction buffer. The cleaved fragments were subjected to synthesis of first-strand cDNA using random hexamer primers and Superscript III reverse transcriptase (Invitrogen). cDNA strands were next synthesized using DNA polymerase I. Overhanging portions of the cDNA duplexes were trimmed to blunt ends and adenylated at the 3′ ends of the DNA fragments. The DNA fragments were ligated with an adaptor containing a hairpin loop and purified. Three microliters of USER Enzyme (NEB) was applied to select cDNA fragments of 150 ~ 200 bp in length and adaptor-ligated before the polymerase chain reaction (PCR). The amplification program was then conducted using high-fidelity DNA polymerase and a universal primer set. The quality of amplicons was assessed using the Agilent Bioanalyzer 2100 system.

Index-coded amplicons were clustered using the cBot Cluster Generation System coupled with a TruSeq PE Cluster Kit v3-cBot-HS (Illumina) following the manufacturer’s instructions. The prepared libraries were sequenced on an Illumina NextSeq 500 platform, and 150 ~ 200-bp paired-end reads were generated.

### Read mapping, transcript annotation, and quantification

Preliminary reads were cleaned by trimming the adapter sequences and removing poly-N or low-quality sequences (Q < 20). The filtered reads were aligned to the mouse reference genome (GRCm37) using the Tophat v2.0.9 program. Tolerance parameters were the default setting to allow mismatches of fewer than two bases. Aligned reads were next subjected to generation of transcriptome assemblies using the Cufflink program. Mutant loci within the assembled transcripts were identified using SAMtools. These transcriptome assemblies generated from individual samples were merged together using the Cuffmerge utility to provide a standard for estimating transcript levels in each condition. Expression levels and the statistical significance of the merged assemblies were calculated using the Cuffdiff analysis.

### Cell culture and differentiation

Mouse C2H10T1/2 fibroblast cells were cultured in Dulbecco’s modified Eagle medium (DMEM; Invitrogen, Carlsbad, CA, USA) supplemented with 10% fetal bovine serum (FBS) (Invitrogen). To induce adipogenesis, C3H10T1/2 cells were shifted to induction medium supplemented with 20% FBS, 0.5 mM IBMX, 12.7 μM dexamethasone, and 10 μg/ml insulin. Forty-eight hours after induction, the induction medium was replaced with differentiation medium (DM) supplemented with 10% FBS and 10 μg/ml insulin, and it was replenished every 2 days.

### Plasmid construction

Expression vectors for the mouse *Nova1* gene were constructed by placing the coding sequence in-frame into the p3XFLAG-CMV14 vector (Sigma, St. Louis, MO, USA). The mice *Nova1* coding region was PCR-amplified using the reverse transcription (RT) product prepared from the total RNA of BATs as the template and then inserted into *Hind* III/*Not* I sites of the vector. pRL-*Nova1* reporters were constructed by inserting the partial region of mouse *Nova1* 3′ untranslated region (UTR) fragments (128 nt) into the pRL-SV40 vector (Promega, Madison, WI, USA). Each fragment of the mouse *Nova1* 3′ UTR was PCR-amplified using a genomic DNA library prepared from mice BATs as the template and then inserted into *Xba* I/*Not* I sites of the pRL-SV40 vector. The mutant pRL-*Nova1* 3′ UTR-F1vector was constructed using the QuikChange site-directed mutagenesis system (Stratagene, Amsterdam, the Netherlands). Sequences of the PCR primer sets are listed in Supplementary Table 1. All constructs were auto-sequenced.

### Transient transfection and RT-PCR analysis

C3H10T1/2 cells were grown to 60% ~ 70% confluence, and the indicated plasmid was transfected using Lipofectamine 3000 following the manufacturer’s protocol (Invitrogen). After 24 h, total RNA and proteins were separately extracted using the Trizol reagent (Invitrogen). For the RT-PCR assay, 1 μg of RNA was reverse-transcribed using SuperScriptase III (Invitrogen) in a 10-μl reaction. The PCR analysis of individual genes was performed using gene-specific primer sets (Supplementary Table 1). The PCR-amplified amplicons of *PKM* and *FGFR2* were then digested with *Pst*I and *EcoR*V to discriminate products containing *PKM2* exon 10 and *FGFR2IIIc* exons. Densities of the PCR products were determined using TotalLab Quant Software. A quantitative (q)RT-PCR was performed with SYBR green fluorescent dye and gene-specific primer sets (Supplementary Table 2) using an ABI One Step™ PCR machine (Applied Biosystems, Foster City, CA, USA). The relative mRNA level was quantitated by the ΔΔ-Ct method, and the level of GAPDH mRNA served as the internal control.

### Immunoblotting assay

The immunoblot analysis was conducted using an enhanced chemiluminescence (ECL) system (Millipore, Billerica, MA, USA), and images were analyzed with the LAS-4000 imaging system (Fujifilm, Tokyo, Japan). Primary antibodies used in this study included polyclonal anti-RBM4 (Santa Cruz Biotechnology, Santa Cruz, CA, USA), monoclonal anti- PTBP2 (Abnova, Taipei, Taiwan), monoclonal anti-PTBP1 (EMD, Millipore), monoclonal anti-Nova1(Abnova), monoclonal anti-Actin (EMD, Millipore), monoclonal anti-FLAG M2 (Sigma-Aldrich, St. Louis, MO, USA), polyclonal anti-ERK1/2, and monoclonal anti-phospho-ERK1/2 (Cell Signaling Technology, Danvers, MA, USA). Intensities of the detected signals were determined using TotalLab Quant Software.

### *In vivo* translation assay

C3H10T1/2 cells were seeded in six-well plates (2 × 10^5^ cells/well) 24 h prior to transfection. The transfection reaction mixture contained 0.5 μg of the pRL-SV40 and engineered Renilla luciferase reporters which contained the partial *Nova1* 3′ UTR fragment, 1 μg of the effector expression vector, and 0.5 μg of the pGL3-basic vector (Promega) as the internal control. After 24 h, transfectants were lysed using passive lysis buffer, and cell debris was removed after centrifugation. Activities of the firefly and Renilla luciferases were measured using a dual-luciferase assay kit (Promega) and the Synergy HT multi-mode microplate reader (BioTek, Winooski, VT, USA).

### RNA electrophoretic mobility shift assay (REMSA)

Recombinant His-tagged PTBP2 was prepared as described previously^14^. RNA probes used were the F1, F2, F3, or mutant F1 elements of the *Nova1* 3′ UTR (128 nt). Partial 3′ UTR elements were *in vitro*-transcribed and used as probes. For RNA-protein interactions, 2 μg of recombinant protein was incubated with 10 nM of the DIG-labeled probe in a 20-μl reaction containing 10 mM HEPES (pH 7.9), 50 μM EDTA, 10% glycerol, 1 mM dithiothreitol, 5 mM MgCl_2_, 0.5 μg/ml bovine serum albumin, and 12.5 ng/ml transfer (t)RNA for 15 min at room temperature. Reactions were analyzed by electrophoresis on an 8% nondenaturing polyacrylamide gel in TBE buffer (45 mM Tris-HCl, 45 mM boric acid, and 1 mM EDTA; pH 8.0). Binding complexes were transferred to nylon membranes (Hybond N, Amersham Bioscience, Piscataway, NJ, USA) that were irradiated under 254-nm light for 60 s. Immunoblotting was conducted by incubating membranes with horseradish peroxidase (HRP)-conjugated anti-DIG Fab fragments (Roche, Mannheim, Germany).

### Mitochondrial respiration assay

A Seahorse XF24 extracellular flux analyzer (Seahorse Bioscience, North Billerica, MA, USA) was used to measure the oxygen consumption rate (OCR; as an indicator of mitochondrial respiration). In brief, 2 × 10^4^ C3H10T1/2 cells were seeded in each well of Seahorse XF24 plates with 250 μl of DMEM and incubated overnight. Prior to the measurement, cells were washed with unbuffered medium and immersed in 675 μl of unbuffered medium without CO_2_ for 1 h. The OCR was assessed in 8-min cycles as recommended by Seahorse Bioscience. The basal and maximal OCRs, and spare respiratory capacity were recorded following injection of complex-specific substrates, including FCCP (2 μM), rotenone (2 μM), and oligomycin (2.5 μg/ml).

### Mitochondria analysis

Proliferating or differentiating C3H10T1/2 cells were subjected to DMEM medium containing100 nM MitoTracker Red FM (Invitrogen) for 45 min at 37 °C. Cells were washed with prewarmed culture medium and visualized with an Olympus IX81 microscope (Olympus, Tokyo, Japan). The signal strength of captured pictures were analyzed with TotalLab Quant Software.

### Oil-red-O staining

Proliferating and differentiating C3H10T1/2 cells were washed twice with phosphate-buffered saline (PBS) and fixed with 4% paraformaldehyde for 60 min at room temperature. Cells were washed with PBS twice and rinsed with 60% isopropanol for 5 min at room temperature. Equilibrated cells were stained with a 0.3% filtered oil-red-O solution (Sigma) for 10 min at room temperature. Stained cells were washed with distilled water three times. For extraction of the oil-red-O dye, the culture dish with absolute isopropanol was shaken at room temperature for 2 h. The extract was centrifuged and analyzed at 550 nm using a NanoDrop 2000 spectrophotometer (Thermo).

### Statistical analyses

Student’s *t*-tests were performed to determine the significance of blot densitometry. *P* < 0.05 was considered statistically significant.

## Additional Information

**How to cite this article**: Lin, J.-C. *et al.* RBM4a-regulated splicing cascade modulates the differentiation and metabolic activities of brown adipocytes. *Sci. Rep.* **6**, 20665; doi: 10.1038/srep20665 (2016).

## Supplementary Material

Supplementary Information

## Figures and Tables

**Table 1:**
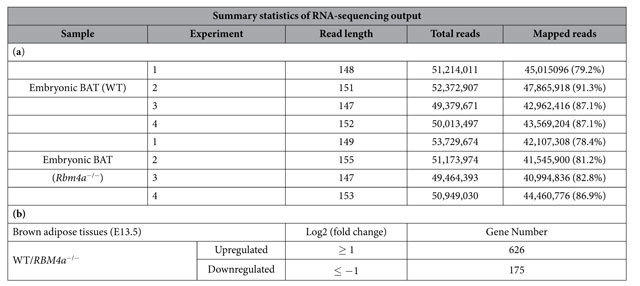
(A) Summary statistics of RNA-sequencing results. (B) The number of differentially expressed genes in WT BATs versus *RBM4a*^−/−^ BATs.

**Table 2:** RNA-binding motif protein 4a (RBM4a) modulates the gene expression profiles in brown adipose tissues.

Differential gene expression in embryonic WT and RBM4a^−/−^ BATs				
Gene ID	Accession No.	Definition	log2 (Fold change)	*p* value
PGC1-β	NM_133249	peroxisome proliferator-activated receptor gamma, coactivator 1 beta	4.999667	0.00005
PGC1-α	NM_008904	peroxisome proliferator-activated receptor gamma, coactivator 1 alpha	3.670846	0.0001
Nova2	NM_001029877	neuro-oncological ventral antigen 2	3.456209	0.0001
UCP1	NM_009463	uncoupling protein 1	1.729614	0.00025
MEF2D	NM_133665	myocyte enhancer factor 2D	1.444777	0.00025
MEF2C	NM_001170537	myocyte enhancer factor 2C	−0.010161	0.001
PTBP2	NM_019550	Polypryrimidine tract binding protein 2	−1.24077	0.0005
RBM4b	NM_025717	RNA binding motif protein 4B	−1.56991	0.00025
Nova1	NM_021361	neuro-oncological ventral antigen 1	−2.10664	0.0001
PTBP1	NM_001077363	Polypryrimidine tract binding protein 1	−2.73061	0.0001

## References

[b1] Nilsen T. W. & Graveley B. R. Expansion of the eukaryotic proteome by alternative splicing. Nature 463, 457–463 (2010). 20110989 10.1038/nature08909 PMC3443858

[b2] Pan Q., Shai O., Lee L. J., Frey B. J. & Blencowe B. J. Deep surveying of alternative splicing complexity in the human transcriptome by high-throughput sequencing. Nat. Genet. 40, 1413–1415 (2008). 18978789 10.1038/ng.259

[b3] Wang E. T. *et al.* Alternative isoform regulation in human tissue transcriptomes. Nature 456, 470–476 (2008). 18978772 10.1038/nature07509 PMC2593745

[b4] Kuwano Y. *et al.* Transformer 2β and miR-204 regulate apoptosis through competitive binding to 3′UTR of BCL2 mRNA. Cell Death Differ. 22, 815–825 (2015). 25342468 10.1038/cdd.2014.176 PMC4392078

[b5] Quesnel-Vallières M., Irimia M., Cordes S. P. & Blencowe B. J. Essential roles for the splicing regulator nSR100/SRRM4 during nervous system development. Genes Dev. 29, 746–59 (2015). 25838543 10.1101/gad.256115.114 PMC4387716

[b6] Matera A. G. & Wang Z. A day in the life of the spliceosome. Nat. Rev. Mol. Cell. Biol. 15, 108–121 (2014). 24452469 10.1038/nrm3742 PMC4060434

[b7] Shi J., Pabon K. & Scotto K. W. Methylxanthines Increase Expression of the Splicing Factor SRSF2 by Regulating Multiple Post-transcriptional Mechanisms. J. Biol. Chem. 290, 14986–5003 (2015). 25818199 10.1074/jbc.M114.624254 PMC4463444

[b8] Taniguchi K. *et al.* Organ-specific PTB1-associated microRNAs determine expression of pyruvate kinase isoforms. Sci. Rep. 27, 8647 (2015). 25721733 10.1038/srep08647 PMC4342556

[b9] Li Y. *et al.* True Sight: a new algorithm for splice junction detection using RNA-seq. Nucleic Acids Res. 41, e51 (2013). 23254332 10.1093/nar/gks1311 PMC3575843

[b10] Giordano A., Smorlesi A., Frontini A., Barbatelli G. & Cinti S. White, brown and pink adipocytes: the extraordinary plasticity of the adipose organ. Eur. J. Endocrinol. 170, R159–171 (2014). 24468979 10.1530/EJE-13-0945

[b11] Cannon B. & Nedergaard J. Brown adipose tissue: Function and physiological significance. Physiol. Rev. 84, 277–359 (2004). 14715917 10.1152/physrev.00015.2003

[b12] Seale P., Kajimura S. & Spiegelman B. M. Transcriptional control of brown adipocyte development and physiological function-of mice and men, Genes Dev. 23, 788–797 (2009). 19339685 10.1101/gad.1779209 PMC2763499

[b13] Rosen E. D. & MacDougald O. A. Adipocyte differentiation from the inside out. Nat. Rev. Mol. Cell Biol. 7, 885–896 (2006). 17139329 10.1038/nrm2066

[b14] Lin J. C., Tarn W. Y. & Hsieh W. K. Emerging role for RNA binding motif protein 4 in the development of brown adipocytes. Biochim. Biophys. Acta. 1843, 7769–7779 (2014). 10.1016/j.bbamcr.2013.12.018 24389249

[b15] Lin J. C. RBM4-MEF2C network constitutes a feed-forward circuit that facilitates the differentiation of brown adipocytes. RNA Biol. 12, 208–220 (2015). 25826570 10.1080/15476286.2015.1017213 PMC4615228

[b16] Lin J. C. & Tarn W. Y. RBM4 down-regulates PTB and antagonizes its activity in muscle cell-specific alternative splicing. J. Cell Biol. 193, 509–520 (2011). 21518792 10.1083/jcb.201007131 PMC3087008

[b17] Lin J. C., Lin C. Y., Tarn W. Y. & Li F. Y. Elevated SRPK1 lessens apoptosis in breast cancer cells through RBM4-regulated splicing events. RNA 20, 1621–1631 (2014). 25140042 10.1261/rna.045583.114 PMC4174443

[b18] Wang Y. *et al.* The splicing factor RBM4 controls apoptosis, proliferation, and migration to suppress tumor progression. Cancer Cell 26, 374–389 (2014). 25203323 10.1016/j.ccr.2014.07.010 PMC4159621

[b19] Lin J. C. *et al.* RBM4 promotes pancreas cell differentiation and insulin expression. Mol. Cell. Biol. 33, 319–327 (2013). 23129807 10.1128/MCB.01266-12 PMC3554116

[b20] Jelen N., Ule J., Zivin M. & Darnell R. B. Evolution of Nova-dependent splicing regulation in the brain. PLoS Genet. 3, 1838–1847 (2007). 17937501 10.1371/journal.pgen.0030173 PMC2014790

[b21] Boutz P. L. *et al.* A post-transcriptional regulatory switch in polypyrimidine tract-binding proteins reprograms alternative splicing in developing neurons. Genes Dev. 21, 1636–1652 (2007). 17606642 10.1101/gad.1558107 PMC1899473

[b22] Vo L. T., Minet M., Schmitter J. M., Lacroute F. & Wyers F. Mpe1, a zinc knuckle protein, is an essential component of yeast cleavage and polyadenylation factor required for the cleavage and polyadenylation of mRNA. Mol. Cell. Biol. 21, 8346–8356 (2001). 11713271 10.1128/MCB.21.24.8346-8356.2001 PMC99999

[b23] Yang Y. Y., Yin G. L. & Darnell R. B. The neuronal RNA-binding protein Nova-2 is implicated as the autoantigen targeted in POMA patients with dementia. Proc. Natl. Acad. Sci. USA 95, 13254–13259 (1998). 9789075 10.1073/pnas.95.22.13254 PMC23773

[b24] Ratti A. *et al.* Post-transcriptional regulation of neuro-oncological ventral antigen 1 by the neuronal RNA-binding proteins ELAV. J. Biol. Chem. 283, 7531–7541 (2008). 18218628 10.1074/jbc.M706082200

[b25] Xu M. & Hecht N. B. Polypyrimidine tract binding protein 2 stabilizes phosphoglycerate kinase 2 mRNA in murine male germ cells by binding to its 3′UTR. Biol. Reprod. 76, 1025–1033 (2007). 17329592 10.1095/biolreprod.107.060079

[b26] Carstens R. P., Wagner E. J. & Garcia-Blanco M. A. An intronic splicing silencer causes skipping of the IIIb exon of fibroblast growth factor receptor 2 through involvement of polypyrimidine tract binding protein. Mol. Cell. Biol. 20, 7388–7400 (2000). 10982855 10.1128/mcb.20.19.7388-7400.2000 PMC86292

[b27] Clower C. V. *et al.* The alternative splicing repressors hnRNP A1/A2 and PTB influence pyruvate kinase isoform expression and cell metabolism. Proc. Natl. Acad. Sci. USA 107, 1894–1899 (2010). 20133837 10.1073/pnas.0914845107 PMC2838216

[b28] Konishi M. *et al.* Role of Fgf receptor 2c in adipocyte hypertrophy in mesenteric white adipose tissue. Mol. Cell Endocrinol. 287, 13–19 (2008). 18396371 10.1016/j.mce.2008.02.010

[b29] Murholm M., Dixen K. & Hansen J. B. Ras signalling regulates differentiation and UCP1 expression in models of brown adipogenesis. Biochim. Biophys. Acta. 1800, 619–627 (2010). 20307629 10.1016/j.bbagen.2010.03.008

[b30] Steinberg Z. *et al.* FGFR2b signaling regulates ex vivo submandibular gland epithelial cell proliferation and branching morphogenesis. Development 132, 1223–1234 (2005). 15716343 10.1242/dev.01690

[b31] Miraoui H. *et al.* Fibroblast growth factor receptor 2 promotes osteogenic differentiation in mesenchymal cells via ERK1/2 and protein kinase C signaling. J. Biol. Chem. 284, 4897–4904 (2009). 19117954 10.1074/jbc.M805432200

[b32] Gao Z. & Cooper T. A. Reexpression of pyruvate kinase M2 in type 1 myofibers correlates with altered glucose metabolism in myotonic dystrophy. Proc. Natl. Acad. Sci. USA 110, 13570–13575 (2013). 23901116 10.1073/pnas.1308806110 PMC3746907

[b33] Chiavarina B. *et al.* Pyruvate kinase expression (PKM1 and PKM2) in cancer-associated fibroblasts drives stromal nutrient production and tumor growth. Cancer Biol. Ther. 12, 1101–1113 (2011). 22236875 10.4161/cbt.12.12.18703 PMC3335944

[b34] Lunt S. Y. *et al.* Pyruvate kinase isoform expression alters nucleotide synthesis to impact cell proliferation. Mol. Cell 57, 95–107 (2015) (2015). 25482511 10.1016/j.molcel.2014.10.027 PMC4289430

[b35] Chang X., Li B. & Rao A. RNA-binding protein hnRNPLL regulates mRNA splicing and stability during B-cell to plasma-cell differentiation. Proc. Natl. Acad. Sci. USA 112, e1888–1897 (2015). 25825742 10.1073/pnas.1422490112 PMC4403190

[b36] Lindholm M. E. *et al.* The human skeletal muscle transcriptome: sex differences, alternative splicing, and tissue homogeneity assessed with RNA sequencing. FASEB J. 28, 4571–4581 (2014). 25016029 10.1096/fj.14-255000

[b37] Makeyev E. V., Zhang J., Carrasco M. A. & Maniatis T. The MicroRNA miR-124 promotes neuronal differentiation by triggering brain-specific alternative pre-mRNA splicing. Mol. Cell 27, 435–448 (2007). 17679093 10.1016/j.molcel.2007.07.015 PMC3139456

[b38] Spellman R., Llorian M. & Smith C. W. Crossregulation and functional redundancy between the splicing regulator PTB and its paralogs nPTB and ROD1. Mol. Cell 27, 420–434 (2007). 17679092 10.1016/j.molcel.2007.06.016 PMC1940037

[b39] Liang Y. C., Lin W. C., Lin Y. J. & Lin J. C. The impact of RNA binding motif protein 4-regulated splicing cascade on the progression and metabolism of colorectal cancer cells. Oncotarget 6, 38046–38060 (2015). 26506517 10.18632/oncotarget.5710 PMC4741983

[b40] Spriggs K. A. *et al.* The human insulin receptor mRNA contains a functional internal ribosome entry segment. Nucleic Acids Res. 37, 5881–5893 (2009). 19654240 10.1093/nar/gkp623 PMC2761284

[b41] Pautz A. *et al.* The polypyrimidine tract-binding protein (PTB) is involved in the post-transcriptional regulation of human inducible nitric oxide synthase expression. J. Biol. Chem. 281, 32294–32302 (2006). 16950790 10.1074/jbc.M603915200

[b42] Oberstrass F. C. *et al.* Structure of PTB bound to RNA: specific binding and implications for splicing regulation. Science 309, 2054–2057 (2005). 16179478 10.1126/science.1114066

[b43] Warzecha C. C., Sato T. K., Nabet B., Hogenesch J. B. & Carstens R. P. ESRP1 and ESRP2 are epithelial cell-type-specific regulators of FGFR2 splicing. Mol. Cell 33, 591–601 (2009). 19285943 10.1016/j.molcel.2009.01.025 PMC2702247

[b44] Asai Y., Yamada K., Watanabe T., Keng V. W. & Noguchi T. Insulin stimulates expression of the pyruvate kinase M gene in 3T3-L1 adipocytes. Biosci. Biotechnol. Biochem. 67, 1272–1277 (2003). 12843653 10.1271/bbb.67.1272

[b45] Colegio O. R. *et al.* Functional polarization of tumour-associated macrophages by tumour-derived lactic acid. Nature 513, 559–563 (2014). 25043024 10.1038/nature13490 PMC4301845

