# Supplementary material for: RBM4a-regulated splicing cascade modulates the differentiation and metabolic activities of brown adipocytes
Source: Sci Rep. 2016 Feb 9;6:20665. doi: 10.1038/srep20665 (PMC4746625; doi:10.1038/srep20665)
Supplement: Supplementary Information [file srep20665-s1.pdf]

RBM4a-regulated splicing cascade modulates the differentiation and metabolic activities of brown adipocytes

Jung-Chun, Lin[1,*], Yi-Han, Lu[1], Yun-Ru, Liu[2], Ying-Ju, Lin[3]

[1] School of Medical Laboratory Science and Biotechnology, College of Medical Science and Technology, Taipei Medical University, Taipei, Taiwan

[2] Joint Biobank, Office of Human Research, Taipei Medical University, Taipei, Taiwan

[3] School of Chinese Medicine, China Medical University, Taichung, Taiwan

Running Title: RBM4a promotes brown adipogenesis

[*]To whom correspondence should be addressed: Jung-Chun Lin

School of Medical Laboratory Science and Biotechnology, College of Medical Science and Technology, Taipei Medical University

250 Wu-Hsing Street, Taipei 11031, Taiwan

Telephone: +8862-27361661 ext. 3330

Fax: +8862-27324510

E-mail: lin2511@tmu.edu.tw

Supplementary Fig. 1

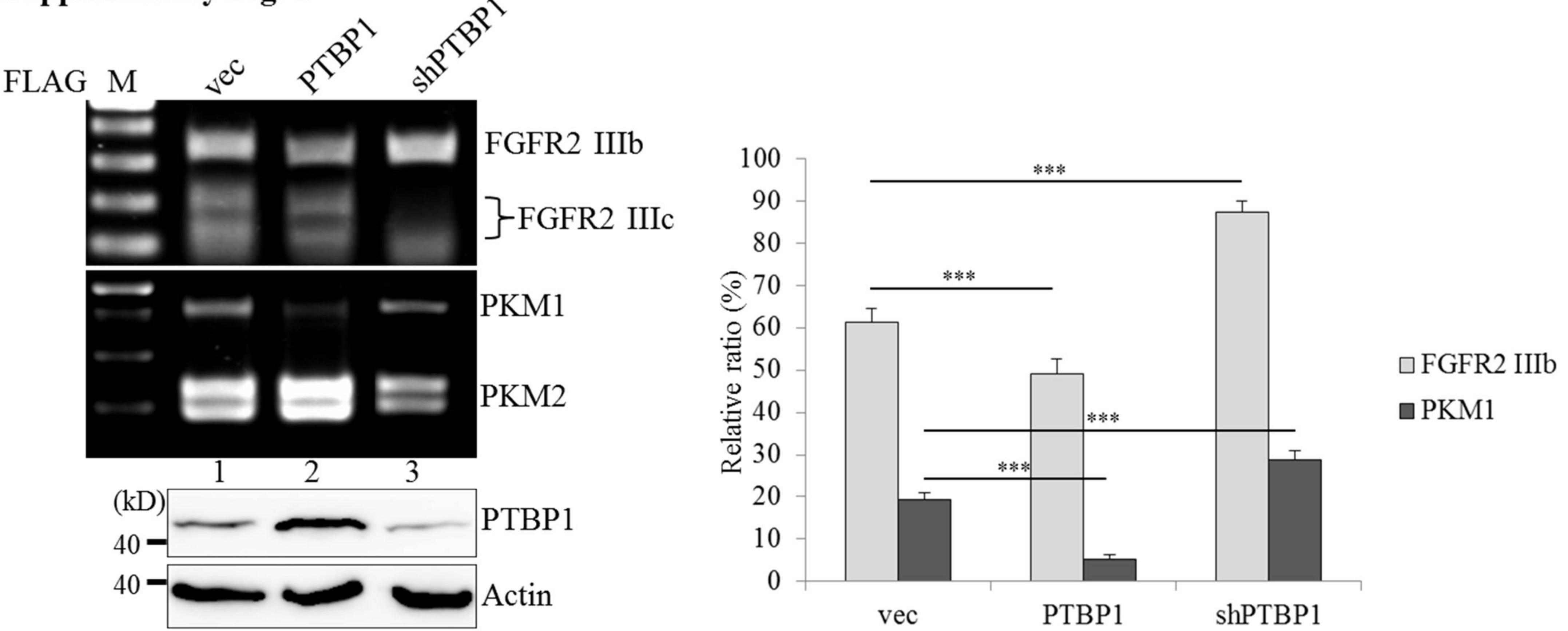

Supplementary Figure 1. PTBP1 modulates the splicing profiles of *FGFR2* and *PKM* genes. Total RNAs and cell extracts were isolated from C3H10T1/2 cells transfected with the expression or targeting vectors of PTBP1, followed by an RT-PCR and immunoblotting analyses with specific primer sets and antibodies.

Supplementary Table 1. PCR Primers.

Gene	Forward	Reverse	cycle
nPTB	gcatttgccaaggagacatcc	cgctgcacatctccataaacac	35
PTB	aagagcagagactacactcga	ctgccgtctgccatctgcacaa	35
Nova1	tctgaccccatgaccacctcca	ctgctgggaaggccgcaaca	35
Nova1 (FL)	atcaagctt atgatggcggcagctccc	atcgcggccgcacccactttctgaggattg	25
Nova1-F1	atctctagagtgtccccattatac	atcgcggccgcatagtcgcattcatttg	35
Nova1-F2	atctctagagttgacaatgtgtatatg	atcgcggccgctaaatcttacactaqgaaac	35
Nova1-F3	atctctagaagcctgccattttac	atcgcggccgctgaccccaccatactcaac	35
Nova1-mF1	gagtgttttaacccctcctttgccccatttggaag	cttccaaatggggcaaaggaggggttaaaacactc	18
UCP1	ctcaggattggcctctacgactc	ttggtgtacatggacatcgca	35
Prdm16	gtcagaggagaaatttgatgg	agaaggaatgctgtgagtag	35
Gapdh	cggagtcaacggatttggtcgtatg	agccttctccatggtggtgaagac	30

Supplementary Table 2. Quantitative PCR Primers.

Gene	Forward	Reverse
UCP1	tacacggggacctacaatgct	ggtcactggaagatatggc
Prdm16	gacattccaatcccaccaga	cacctctgtatccgtcagca
Gapdh	cggagtcaacggatttggtcgtatg	agccttctccatggtggtgaagac
